# The diagnostic value of global longitudinal strain on doxorubicin-induced cardiotoxicity in pediatric cancer patients: a cross-sectional study

**DOI:** 10.3389/fped.2025.1615563

**Published:** 2025-11-07

**Authors:** Dyah Ayu Shinta Lesmanawati, Dany Hilmanto, Nur Melani Sari, Dzulfikar D. L. Hakim, Sri Endah Rahayuningsih, Susi Susanah

**Affiliations:** 1 Department of Child Health, Hasan Sadikin General Hospital, Faculty of Medicine, Universitas Padjadjaran, Bandung, Indonesia; 2 Nephrology Division, Department of Child Health, Hasan Sadikin General Hospital, Faculty of Medicine, Universitas Padjadjaran, Bandung, Indonesia; 3 Hematology-Oncology Division, Department of Child Health, Hasan Sadikin General Hospital, Faculty of Medicine, Universitas Padjadjaran, Bandung, Indonesia; 4 Pediatric Emergency and Intensive Care Division, Department of Child Health, Hasan Sadikin General Hospital, Faculty of Medicine, Universitas Padjadjaran, Bandung, Indonesia; 5 Cardiology Division, Department of Child Health, Hasan Sadikin General Hospital, Faculty of Medicine, Universitas Padjadjaran, Bandung, Indonesia

**Keywords:** pediatric cancer, doxorubicin, left ventricular dysfunction, speckle tracking echocardiography, cardiotoxicity

## Abstract

**Background:**

Chemotherapy is a critical modality in the treatment of pediatric cancer. However, the risk of adverse effects remains a significant challenge despite the considerable advancements in chemotherapy efficacy. Anthracyclines are a cornerstone in the treatment of various childhood cancers but are associated with a notable risk of cardiotoxicity. Early detection of cardiac dysfunction in pediatric cancer patients is vital to prevent progression to heart failure, with echocardiography being one of the primary modalities for such assessments. Speckle Tracking Echocardiography (STE) is a recommended method for the early detection of cardiotoxicity in adult cancer patients, although its application in the pediatric population remains limited. This study aims to assess the diagnostic value of global longitudinal strain (GLS) in detecting doxorubicin-induced cardiotoxicity in pediatric cancer patients and to examine its correlation with cumulative doxorubicin doses.

**Methods:**

This cross-sectional study involved 52 pediatric cancer patients at Dr. Hasan Sadikin General Hospital. We performed both conventional echocardiography and STE for those receiving doxorubicin as their chemotherapy regimen. STE evaluated the correlation between cumulative doxorubicin dose and left ventricular function. Receiver Operating Characteristic (ROC) and area under the curve (AUC) were performed to evaluate the predictive model of GLS in diagnosing cardiac dysfunction based on conventional methods. This analysis also measures the model of cumulative doxorubicin doses to predict reduced GLS percentages.

**Results:**

A total of 52 subjects (67.3% male, mean age 10.3 years) were included in this study. Conventional echocardiography detected systolic dysfunction in 21.2% and diastolic dysfunction in 23.1% of patients. GLS measurements identified ventricular dysfunction in 19.2% of subjects. Significant risk factors for cardiotoxicity were cancer type and nutritional status, with *p*-values of 0.006 and 0.028, respectively. GLS scores were significantly lower in patients with low systolic function (18.3%) compared to those with normal function (−23.6%), with a mean difference of 5.3% (*p* < 0.0001), and a similar difference was found for diastolic dysfunction. ROC curve analysis revealed an area under the curve (AUC) of 0.793 for systolic dysfunction and 0.746 for diastolic dysfunction, with cutoff values of −19.10 for systolic (72.7% sensitivity, 65.4% specificity) and −19.55 for diastolic dysfunction (58.3% sensitivity, 87.5% specificity).

**Conclusion:**

This study suggests that GLS derived from STE may help identify early alterations in left ventricular function in pediatric patients receiving doxorubicin. Although reduced GLS was associated with systolic and diastolic dysfunction, differences were modest and within physiological limits. These findings highlight the potential role of GLS in cardiac monitoring, though further research is needed to confirm its diagnostic value.

## 1 Introduction

The remarkable improvement in survival rates for children with cancer, which has risen from 30% in the 1960s to over 80% today, is a significant achievement of the 21st century (1). This success has been primarily due to advances in treatment regimens, particularly the use of doxorubicin, a highly effective chemotherapy drug for various pediatric cancers, including osteosarcoma, lymphoma, and leukemia. However, the increasing survival rates bring forth the challenge of managing the long-term adverse effects of cancer therapies, particularly cardiotoxicity induced by doxorubicin (2).

Doxorubicin's cardiotoxicity is a well-documented concern, linked to mechanisms such as oxidative stress, mitochondrial dysfunction, and DNA damage in heart cells, leading to potential heart failure. While acute cardiotoxicity is typically reversible, chronic effects, including left ventricular dysfunction, can emerge months or years after therapy, underscoring the need for early detection (3). Traditionally, the cumulative dose of doxorubicin is used to assess the risk of cardiotoxicity, with a threshold of 300 mg/m^2^ recommended to minimize the risk of heart failure (4). However, even doses below this limit can cause subclinical myocardial damage that may not be detectable with conventional methods.

Current surveillance relies on two-dimensional echocardiography, which assesses heart function via ejection fraction and fractional shortening but is less sensitive in detecting early myocardial changes (5).

Speckle Tracking Echocardiography (STE) has emerged as a more sensitive tool. By measuring myocardial strain, it enables the detection of subtle alterations in myocardial function (6). Global Longitudinal Strain (GLS), a key parameter derived from STE, is particularly valuable in pediatric oncology, as it can detect both systolic and diastolic dysfunction before changes in ejection fraction occur, making early intervention possible (7).

The early detection of left ventricle dysfunction in children with cancer is of particular interest, as it can serve as a sensitive indicator of doxorubicin-induced cardiotoxicity. Our study aims to evaluate the use of STE in detecting left ventricle dysfunction in pediatric cancer patients. The goal is establishing early intervention thresholds, ultimately improving this vulnerable population's long-term cardiac health.

## 2 Materials and methods

### 2.1 Study design

This observational analytic study employed a cross-sectional design to investigate the correlation between cumulative doxorubicin doses and the onset of cardiotoxicity in pediatric cancer patients. Data on demographics and baseline laboratory characteristics were gathered from medical records, while clinical assessments and echocardiographic evaluations were performed directly on the patients. The study targeted pediatric cancer patients receiving doxorubicin therapy at RSUP Dr. Hasan Sadikin, Bandung, with cancer diagnoses confirmed through clinical and pathological criteria.

This observational analytic study, employing a cross-sectional design, investigated the correlation between cumulative doxorubicin doses and the onset of cardiotoxicity in pediatric cancer patients at RSUP Dr. Hasan Sadikin, Bandung, with cancer diagnoses confirmed through clinical and pathological criteria. Demographic data, baseline laboratory characteristics, and echocardiographic evaluations were collected from medical records and direct patient assessments. All patients received doxorubicin as part of their chemotherapy regimen. The cumulative dose of doxorubicin was documented for each patient and considered in the interpretation of cardiac function outcomes, particularly in relation to global longitudinal strain (GLS) values.

Eligible participants were children aged 1 month to <18 years, diagnosed with cancer through methods like bone marrow aspiration or histopathology, and undergoing chemotherapy with doxorubicin. Informed consent was required for participation. Exclusion criteria included congenital or acquired heart disease, valvular abnormalities, prior heart failure, pericardial effusion, and genetic syndromes such as Down, Turner, or Noonan syndromes. Patients who were immobile or had incomplete medical records were also excluded. The sample size was calculated to be at least 50 participants, determined using correlation and cross-sectional study formulas, with consecutive sampling used to reach the target size.

Data were collected via an electronic case report form (CRF), integrating information from medical records, chemotherapy regimens, and echocardiographic evaluations conducted at RSUP Dr. Hasan Sadikin. Ethical approval was obtained prior to the study, which proceeded until the desired sample size was achieved. Screening for eligible subjects was done through medical records, and informed consent was obtained from both patients and their families before enrolment. Demographic and relevant clinical data were extracted, and Speckle Tracking Echocardiography (STE) was employed for further assessments. Statistical analysis was performed after data collection to thoroughly explore the study's objectives.

### 2.2 Echocardiographic assessment

Echocardiographic examinations were performed using a Philips EPIQ CVx ultrasound system (Philips Medical Systems, Andover, MA, USA), equipped with pediatric transducers and advanced cardiac imaging capabilities, including STE. All echocardiographic studies were conducted by SE, a pediatric cardiology consultant with subspecialty training in cardiac imaging, who was blinded to the patients' cumulative anthracycline exposure and clinical outcomes. The examinations were performed with the child in the left lateral decubitus position, in a quiet and temperature-controlled room to minimize motion artifacts. The echocardiographic protocol included standard 2D and M-mode imaging, obtained from parasternal long- and short-axis views, and apical 4-chamber, 2-chamber, and long-axis views to assess LV structure and function. Left Ventricular EF was measured using the biplane Simpson's method. Speckle-tracking echocardiography (STE) was performed using vendor-specific software integrated into the Philips CVx system. Global longitudinal strain (GLS) was measured from the apical views, with the average of the three views used as the final GLS value. All images were acquired at high frame rates (60–90 frames per second), and at least three consecutive cardiac cycles were recorded during quiet respiration or breath-hold when feasible. Offline strain analysis was conducted by a single trained observer to reduce variability. Subclinical cardiac dysfunction was defined as a GLS value greater than −19%. The −19% GLS threshold was selected based on current pediatric echocardiographic literature. A systematic review and meta-analysis, reported that normal GLS values in healthy children generally fall between −19% and −22% (8). A GLS value less negative than −19% may therefore indicate early or subclinical systolic dysfunction. This threshold is further supported by Chatterjee et al. (9), who recommended −19% as a reasonable cut-off when comparing GLS to conventional parameters such as LVEF and FS. In addition to the published evidence, this cut-off value was also determined in consultation with pediatric cardiology experts at the study site through consensus, taking into account both current evidence and their clinical judgment in managing pediatric patients at risk for cardiotoxicity.

### 2.3 Statistical analysis

The analysis incorporated both descriptive and inferential statistical methods. Descriptive analysis was performed to summarize subject characteristics and study outcomes, using tables to present the data. Numerical variables were expressed as means with standard deviations (SD) or medians with interquartile ranges (IQR), depending on the distribution assessed by the Kolmogorov–Smirnov test.

Categorical variables were summarized as frequencies and percentages.

Inferential analysis evaluated associations between risk factors and left ventricular dysfunction. Chisquare tests were utilized to identify certain risk factors, while Receiver Operating Characteristic (ROC) curves and Area Under the Curve (AUC) analyses were conducted to assess to evaluate the predictive model of GLS in diagnosing cardiac dysfunction based on conventional methods. This analysis also measures the model of cumulative doxorubicin doses to predict reduced GLS percentages. The Youden Index was applied to determine the optimal threshold for prediction.

All statistical analyses were performed using SPSS v26 software. A *p*-value of <0.05 was considered statistically significant, with results reported within a 95% confidence interval. These methods ensured robust evaluation of both descriptive patterns and inferential associations, facilitating accurate interpretation of study outcomes.

## 3 Result

### 3.1 Demographic and clinical characteristics of study subjects

This study was conducted from November 2023 to August 2024, focusing on the correlation between the cumulative dose of doxorubicin in pediatric cancer therapy and left ventricular function assessed using speckle tracking echocardiography (STE). Fifty-two subjects met the inclusion criteria, with diagnoses and treatment regimens determined by pediatric hematology consultants at the Department of Pediatrics, Faculty of Medicine, Universitas Padjadjaran/Dr. Hasan Sadikin Hospital.

The characteristic of the study subject presented in Supplementary Table S1 above. The majority of subjects were male (67.3%) with a mean age of 10.3 years (SD: 5.1), predominantly within the 6–13 years age group (42.3%). The most common cancer type was acute myeloid leukemia (AML) (34.6%), and most subjects had undergone fewer than three chemotherapy cycles (53.8%). Normal nutritional status was observed in 78.8% of the subjects, with a mean BMI of 16.7 kg/m^2^ (SD: 4.0). Anemia was present in 73.1% of the patients.

### 3.2 Cardiac function examination results

#### 3.2.1 Systolic function

Supplementary Table S2 indicated the echocardiographic assessment revealed normal left ventricular ejection fraction (LVEF) in 82.7% of subjects, with 17.3% experiencing low LVEF (mean: 64.3%). Fractional shortening (FS) was normal in 80.8% of the subjects, with 19.2% showing abnormal values (mean: 34.3%). The myocardial performance index (MPI) or Tei Index had a mean value of 0.58 (SD: 0.11), with 44.2% of patients having normal values. Global longitudinal strain (GLS) was used to assess global myocardial function. Most subjects (80.8%) had normal GLS values (≥ −19), while 19.2% exhibited GLS below reference range (< −19), with a mean GLS of −22.5%.

#### 3.2.2 Diastolic function

Supplementary Table S3 shows the parameters of diastolic dysfunction. The mean septal E/E’ ratio was 9.1, with 94.2% of the subjects having normal values and 5.8 showing abnormal values. All subjects had normal lateral E/E' ratios (mean: 6.3). The mean E' septal velocity was 10.7 cm/s, with 88.5% of subjects showing normal values, while 11.5% had abnormal E' septal values. The mean E' lateral velocity was 15.5 cm/s, with all subjects showing normal values. Left ventricular structural parameters included a mean posterior wall dimension of 6.7 mm (SD: 1.5) during diastole and 10.8 mm (SD: 2.0) during systole. The mean left ventricular mass index (LVMI) was 81.4 g/m^2^ (SD: 48.9).

Early signs of ventricular dysfunction were detected in asymptomatic subjects through echocardiography. Systolic dysfunction, indicated by LVEF <55%, FS <28%, or Tei Index >0.5, was observed in 61.5% of subjects. Diastolic dysfunction, defined by abnormal E/A ratio, E/E' > 14, or E’ septal <7 cm/s, was observed in 23.1% of subjects.

### 3.3 Risk factor analysis of left ventricular dysfunction

Risk factors such as age, sex, chemotherapy cycles, anemia status, and nutritional status were evaluated using bivariate analysis as stated in Supplementary Table S4 above. Among these, cancer type (*p* = 0.006) and nutritional status (*p* = 0.028) were statistically significant predictors of left ventricular dysfunction.

### 3.4 The role of GLS in early detection of ventricular dysfunction

Supplementary Table S5 compares mean GLS scores between patients with low systolic and those with normal systolic function, as assessed by conventional echocardiography. The mean difference in GLS scores between the two groups was 5.3% (95% CI: 2.5–8.0). This difference was statistically significant, with a *p*-value of <0.0001.

Supplementary Table S6 highlights the mean GLS scores for low and normal diastolic function patients. The observed mean difference between the two groups was 5.3% (95% CI: 2.5–8.0). This finding is highly statistically significant (*p* < 0.0001).

**Figure 1:**
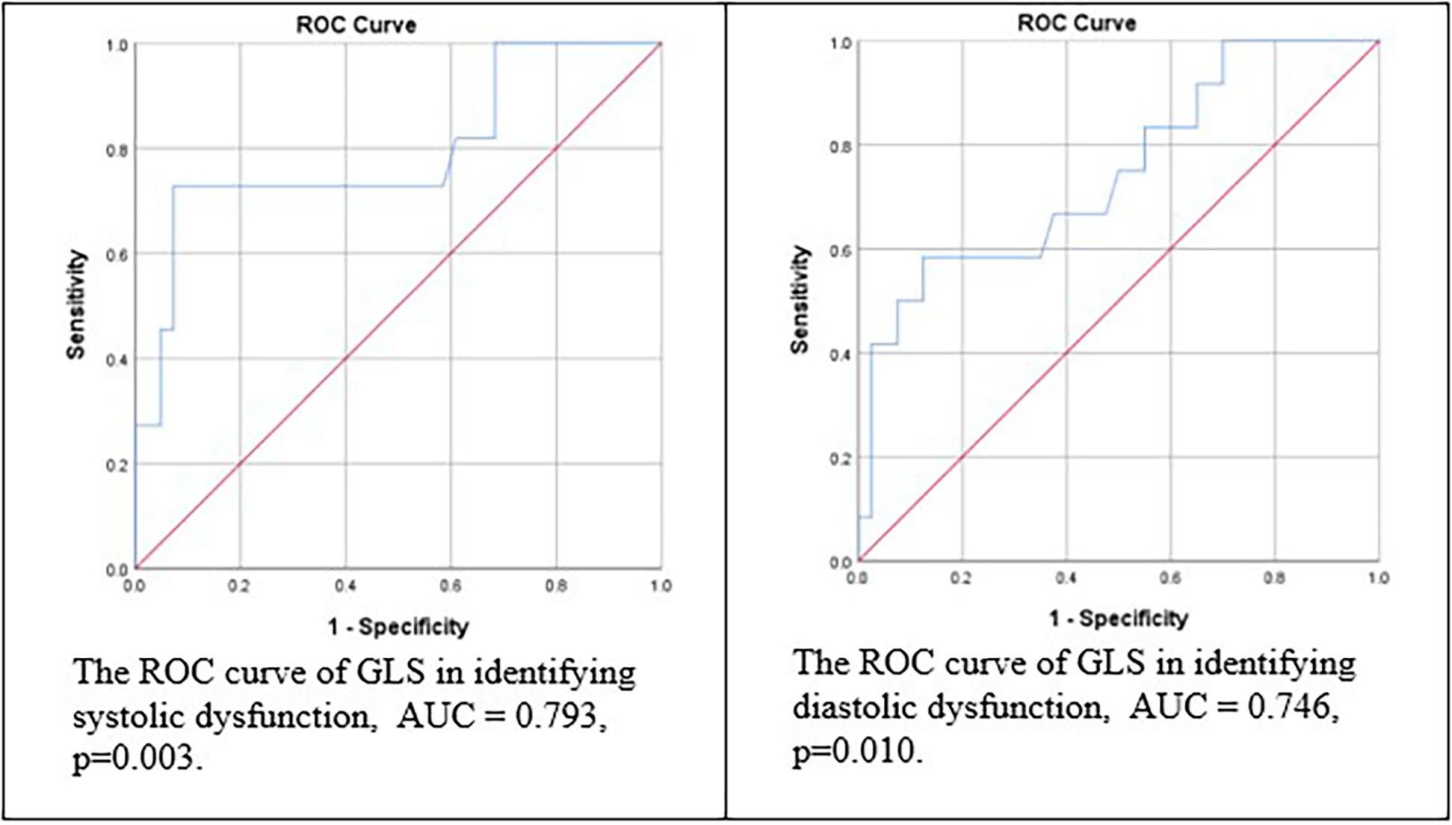
The ROC and AUC of GLS in identifying left ventricle dysfunction.

The area under the ROC curve for the prediction of LV systolic dysfunction is depicted in Figure 1 and Supplementary Table S7. GLS had an area under the curve of 0.793 for systolic function and 0.746 for diastolic function (Figure 1). The ROC curve was constructed using GLS, which gave a cut-off value of (−19.10) with 72.7% sensitivity and 65.4% for systolic function, while the cut-off value of diastolic function was (19.55) with 58.3% sensitivity and 87.5% specificity for the prediction of LV dysfunction in children receiving doxorubicin chemotherapy. This value indicated that GLS is a good predictor for LV dysfunction.

## 4 Discussion

### 4.1 Demographic and clinical characteristics of the subject

The study included a majority of male participants (67.3%), with an average age of 10.3 years, consistent with several epidemiological studies on pediatric cancer showing a higher incidence of leukemia in males than in females (10). According to research by Kachuri, Linda, et al., the incidence of leukemia is higher in male children, possibly due to hormonal factors or genetic susceptibility (11). Acute Myelogenous Leukemia (AML) was the most common cancer, representing 34.6% of cases, which aligns with global studies that show leukemia is one of the most common hematological malignancies in children, although AML is less common than Acute Lymphoblastic Leukemia (ALL) (12). Most participants had undergone fewer than three chemotherapy cycles (53.8%), correlating with the cumulative doxorubicin dose and the increased likelihood of cardiotoxicity (13).

Interestingly, 78.8% of subjects had normal nutritional status, with a mean BMI of 16.7 kg/m^2^, despite previous studies suggesting that pediatric cancer patients often experience malnutrition due to treatment side effects and metabolic disturbances (14). Good nutritional status is critical to influencing treatment tolerance and recovery. Studies show that it contributes to better chemotherapy response and clinical outcomes (15).

Anemia was present in 73.1% of patients, a factor complicating chemotherapy-induced cardiotoxicity (16). Anemia can result from chemotherapy, cancer cell infiltration into the bone marrow, or systemic inflammatory responses. It leads to hypoxia, reducing myocardial oxygenation and causing mitochondrial damage, ultimately harming heart cells. Additionally, increased cardiac output in anemic patients can contribute to accelerated ventricular remodeling (17).

### 4.2 Assessment of left ventricular function

Left ventricular function was assessed using conventional echocardiographic techniques, evaluating ejection fraction (EF), fractional shortening (FS), and the Myocardial Performance Index (MPI). Conventional echocardiography assumes a cylindrical shape for the ventricle, which may lead to underestimation or overestimation of values. However, Speckle Tracking Echocardiography (STE) and Global Longitudinal Strain (GLS) have become preferred methods for evaluating left ventricular function (18). In this study, most subjects had normal ventricular function, with a small subset showing subclinical abnormalities. Specifically, 17.3% had EF <55%, while 19.2% had FS <28%, indicating systolic dysfunction, often associated with myocardial adaptation changes due to increased hemodynamic load.

Diastolic function was assessed using E’, E/E', and Left Atrial Volume Index (LAVI). The septal E/E' ratio was normal for most subjects, with 88.5% showing normal septal E' velocity, while 11.5% had abnormal values indicating impaired ventricular relaxation. All subjects had normal lateral E' velocities and E/E’ lateral ratios. Only 1.9% had elevated LAVI, suggesting that diastolic dysfunction was generally mild. These findings support the sensitivity of E' velocity and the E/E' ratio as early indicators of diastolic dysfunction, even in the absence of clear systolic abnormalities. Previous studies have supported the idea that E' velocity and the E/E' ratio are sensitive markers for detecting left ventricular relaxation abnormalities, which often precede more significant diastolic dysfunction. According to Nagueh et al., E', E/E’, and LAVI parameters can serve as early indicators of diastolic dysfunction, even in the absence of clear systolic abnormalities (19).

The MPI evaluation revealed abnormal values in 55.8% of subjects, suggesting mild to moderate combined systolic and diastolic dysfunction. Additionally, STE with GLS detected early longitudinal contractility abnormalities, which may be undetected by EF alone. These findings align with studies by Shitole et al., and Sangweni et al., which highlight that subclinical changes detected by MPI and GLS in patients who appear normal may indicate more significant myocardial damage in the long term, potentially leading to clinical heart dysfunction (3, 20).

### 4.3 Risk factors analysis of left ventricular dysfunction

Pediatric cancer patients undergoing doxorubicin therapy are influenced by various factors that affect the incidence of left ventricular dysfunction. Statistically significant risk factors identified in this study were cancer type and nutritional status. As supported by previous studies, specific cancer types have a higher risk for cardiotoxicity. Dong et al. found that solid tumors such as osteosarcoma pose a higher risk for early ventricular dysfunction, as the doses given are generally higher than those for hematological malignancies, increasing cumulative doxorubicin exposure (21). Additionally, severe malnutrition has been associated with an increased risk of cardiovascular dysfunction in cancer patients. Research by Gándara-Mireles et al. showed that malnutrition exacerbates the toxic effects of cancer therapy on heart function through inflammatory mechanisms and increased oxidative stress (22). Cardinale et al. identified age, cancer type, number of chemotherapy cycles, and nutritional status as key predictors for cardiotoxicity in cancer patients (23).

### 4.4 The role of speckle tracking echocardiography in early detection of cardiotoxicity

Speckle Tracking Echocardiography (STE) using GLS measurements has potential as a tool for early detection of left ventricular dysfunction in patients undergoing doxorubicin therapy. In this study, patients with low GLS had three times the likelihood of systolic dysfunction and nine times the likelihood of diastolic dysfunction compared to those with normal GLS values. This finding aligns with several previous studies indicating that GLS is a sensitive tool for detecting subclinical ventricular dysfunction before EF declines. Sangweni et al. demonstrated that GLS changes could detect heart function impairment at the subclinical stage, enabling earlier detection of cardiotoxicity than EF alone (3). The Global Cardio-Oncology Society also recommends STE as part of cardiac monitoring for patients receiving anthracycline therapy due to its ability to detect early functional changes associated with drug toxicity (24). However, STE has limitations related to equipment availability and operator skills. Therefore, many studies still consider LVEF as the gold standard for detecting systolic dysfunction, as it is more widely integrated into clinical protocols (3, 25). A combination of STE and conventional methods like LVEF represents a more holistic approach for detecting and monitoring doxorubicin-induced cardiotoxicity in pediatric cancer patients.

## 5 Strengths and limitations

This study has several strengths and limitations that merit consideration. One of the main strengths lies in its pioneering approach as the first study in Indonesia focusing on early detection of left ventricular dysfunction using STE in pediatric populations. By utilizing GLS parameters, the research highlights the potential of advanced imaging techniques in identifying subclinical cardiac dysfunction before clinical symptoms arise. This contributes significantly to the growing body of evidence supporting STE's utility in pediatric oncology.

However, there are notable limitations. First, the study population only included clinically stable and transportable patients, excluding those in more severe conditions. This selection criterion introduces potential selection bias and may limit the generalizability of the findings. Second, the study employed a cross-sectional design with single-time-point measurements, precluding the evaluation of longitudinal changes in cardiac function or the dynamic effects of cumulative doxorubicin exposure over time. Third, the majority of participants were in the early phases of chemotherapy, and the findings may not reflect the cumulative cardiotoxic effects in later treatment stages. Fourth, the absence of a control group without doxorubicin therapy restricts the ability to discern specific impacts attributable solely to doxorubicin. Fifth, while all patients received doxorubicin, a subset also received other chemotherapeutic agents with known cardiotoxic potential, such as cyclophosphamide and ifosfamide. This co-exposure may have influenced cardiac function independently of doxorubicin, thereby introducing potential confounding in the interpretation of GLS values. Moreover, as all measurements were taken post-chemotherapy, it is not possible to determine whether GLS values below reference range reflect true changes from an individual's baseline.

Despite these limitations, the study establishes a foundational understanding and underscores the need for further research, particularly longitudinal studies with diverse patient cohorts and control groups with stratified analyses by treatment protocol, to comprehensively evaluate the long-term implications of doxorubicin-induced cardiotoxicity in children.

## 6 Conclusion

This study provides preliminary evidence that STE, particularly GLS, may have a role in identifying early changes in left ventricular function among pediatric cancer patients receiving doxorubicin. Although some associations were observed between reduced GLS and systolic or diastolic dysfunction, the differences remained modest and largely within physiologic variation. As such, GLS should be interpreted cautiously and in conjunction with conventional echocardiographic parameters. While these findings suggest potential utility of GLS in cardiac monitoring, further research with larger cohorts, baseline measurements, and longitudinal follow-up is needed to more definitively determine its diagnostic value in detecting early cardiotoxicity.

## Data Availability

The data analyzed in this study is subject to the following licenses/restrictions: The dataset contains sensitive patient information and is not publicly available due to privacy and ethical restrictions. Access to the data may be granted upon reasonable request and with permission from the Research Ethics Committee of Dr. Hasan Sadikin General Hospital, Bandung, Indonesia. Requests to access these datasets should be directed to Sri Endah Rahayuningsih, sri.endah@unpad.ac.id.
