# Supplementary material for: The diagnostic value of global longitudinal strain on doxorubicin-induced cardiotoxicity in pediatric cancer patients: a cross-sectional study
Source: Front Pediatr. 2025 Nov 7;13:1615563. doi: 10.3389/fped.2025.1615563 (PMC12634503; doi:10.3389/fped.2025.1615563)
Supplement: Supplementary file 1 [file Datasheet1.docx]

**Supplementary Material**

**Table 1. Demographics and characteristics of the participants**

| Characteristics | Mean (+ SD) | N (%) |
| --- | --- | --- |
| Age at recruitment, years | 10,3 (5,1) |  |
| Age at recruitment, years   - 0-5 years - 6-13 years - 14-18 years |  | 11 (21,2)  22 (42,3)  19 (36,5) |
| Gender   - Male - Female |  | 35 (67,3)  17 (32,7) |
| Weight (kg) | 28,3 (15,4) |  |
| Height (cm) | 125,0 (29,2) |  |
| BMI (kg/m2) | 16,7 (4,0) |  |
| Nutritional state   - Severe malnutrition - Mild-moderate malnutrition - Normal - Overweight |  | 6 (11,5)  4 (7,7)  41 (78,8)  1 (1,9) |
| Cancer types   - Nephroblastoma - Osteosarcoma - Hepatoblastoma - Acute Myeloblastic Leukemia - Hodgkin Lymphoma - Ewing Sarcoma - Neuroblastoma - Pleuropulmonary Blastoma |  | 5 (9,6)  10 (19,2)  3 (5,8)  18 (34,6)  7 (13,5)  7 (13,5)  1 (1,9)  1 (1,9) |
| Chemotherapy cycle   - <3 - >=3 |  | 28 (53,8)  24 (46,2) |
| Blood pressure (mmHg)   - Systolic - Diastolic | 96,8 (16,2)  66,9 (10,2) |  |
| Oxygen saturation (%) | 96,9 (1,2) |  |
| Pulse (beat/min) | 97,9 (10,9) |  |
| Anemia   - Yes - No |  | 38 (73,1)  14 (26,9) |
| Cumulative dose (mg) | 215,9 (269,6) |  |

Table 2. Left Ventricle Systolic Function of the 52 Participants

| Parameters | Mean (+ SD) | N (%) |
| --- | --- | --- |
| Ejection Fraction (%)   - <55% - >55% | 64,3 (9,7) | 9 (17,3)  43 (82,7) |
| Fractional Shortening (%)   - <28% - >28% | 34,3 (7,8) | 10 (19,2)  42 (80,8) |
| CO (L/min) | 3770,2 (1496,2) |  |
| CI (L/min/m2) | 4162,3 (1584,6) |  |
| MPI   - Normal - Abnormal | 0,58 (0,11) | 23 (44,2)  29 (55,8) |
| IVRT (ms) |  | 70,4 (31,0) |
| LV GLS (%)   - < (-19) - >(-19) | 22,5 (4,5) | 10 (19,2)  42 (80,8) |

Table 3. Left Ventricle Diastolic Function of the 52 Participants

| Parameter | Mean (+ SD) | N (%) |
| --- | --- | --- |
| Peak E (cm/s) | 0,94 (0,22) |  |
| Peak A (cm/s) | 0,66 (0,21) |  |
| Ratio E/A | 1,5 (0,6) |  |
| DT (ms) | 106,5 (40,5) |  |
| LA vol index   - <34 - >34 | 15,4 (11,0) | 51 (98,1)  1 (1,9) |
| A’ septal (cm/s) | 6,4 (1,8) |  |
| S’ septal (cm/s) | 0,09 (0,06) |  |
| E’ septal   - <7 - >7 | 10,7 (2,9) | 6 (11,5)  46 (88,5) |
| Ratio E/E’ septal   - <14 - >14 | 9,1 (2,6) | 49 (94,2)  3 (5,8) |
| A’ lateral (cm/det) | 7,1 (2,2) |  |
| S’ lateral (cm/det) | 0,10 (0,02) |  |
| E’ lateral   - <10 - >10 | 15,5 (3,6) | 0 (0,0)  52 (100,0) |
| Ratio E/E’ lateral   - <14 - >14 | 6,3 (1,8) | 52 (100,0)  0 (0,0) |
| S PV (cm/s) | 53,0 (17,9) |  |
| D PV (cm/s) | 55,3 (13,3) |  |
| Ratio SD | 0,99 (0,35) |  |

Table 4. Chi-square analysis for risk factors of left ventricle dysfunction

| Variable | LV-GLS N (%) | | P |
| --- | --- | --- | --- |
|  | Below Reference Range | Normal |  |
| Age at recruitment, years   - 0-5 years - 6-13 years - 14-18 years | 2 (18,0)  5 (50,0)  3 (30,0) | 9 (21,4)  17 (40,5)  16 (38,1) | 0,85 |
| Gender   - Male - Female | 8 (80,0)  2 (20,0) | 27 (64,3)  15 (35,7) | 0,46 |
| Cancer types   - Nephroblastoma - Osteosarcoma - Hepatoblastoma - Acute Myeloblastic Leukemia - Hodgkin Lymphoma - Ewing Sarcoma - Neuroblastoma - Pleuropulmonary Blastoma | 0 (0,0)  1 (10,0)  3 (30,0)  1 (10,0)  2 (20,0)  2 (20,0)  1 (10,0)  0 (0,0) | 5 (11,9)  9 (21,4)  0 (0,0)  17 (40,5)  5 (11,9)  5 (11,9)  0 (0,0)  1 (2,4) | 0,006 |
| Chemotherapy cycle   - <3 - >=3 | 5 (50,0)  5 (50,0) | 23 (54,8)  19 (45,2) | 0,78 |
| Anemia   - Yes - No | 8 (80,0)  2 (20,0) | 30 (71,4)  12 (28,6) | 0,71 |
| Nutritional state   - Severe malnutrition - Mild-moderate malnutrition - Normal - Overweight | 4 (40,0)  0 (0,0)  6 (60,0)  0 (0,0) | 2 (4,8)  4 (9,5)  35 (83,3)  1 (2,4) | 0,028 |

Table 5. The difference in the mean GLS scores among patients with systolic dysfunction (assessed by conventional echocardiography)

| Variable | Systolic Function | | Mean Difference  (95% CI) | p-value |
| --- | --- | --- | --- | --- |
|  | Below Reference Range | Normal |  |  |
| Global Longitudinal Strain (%) | -18,3 | -23,6 | 5,3 (2,5-8,0) | <0,0001 |

Table 6. The difference in the mean GLS scores among patients with diastolic dysfunction (assessed by conventional echocardiography)

| Variable | Diastolic Function | | Mean Difference  (95% CI) | p-value |
| --- | --- | --- | --- | --- |
|  | Below Reference Range | Normal |  |  |
| Global Longitudinal Strain (%) | -18,8 | -23,5 | 4,7 (2,0-7,4) | 0,001 |

Table 7. The ROC and AUC of GLS in identifying left ventricle dysfunction (assessed by conventional echocardiography)

| Parameters | Global Longitudinal Strain | | | |
| --- | --- | --- | --- | --- |
|  | Cut off | Sensitivity (%) | Specificity (%) | p-value |
| Systolic | -(19.10) | 72.7 | 65.4 | 0.003 |
| Diastolic | -(19.55) | 58.3 | 87.5 | 0.010 |

Table 8. Individual data of patients’ echocardiographic values

| **Patient ID** | **LVEF (%)** | **FS (%)** | **GLS (%)** |
| --- | --- | --- | --- |
| 001 | 61.80 | 31.80 | 26.70 |
| 002 | 66.20 | 35.40 | 27.40 |
| 003 | 45.10 | 22.20 | 18.70 |
| 004 | 68.50 | 37.20 | 19.90 |
| 005 | 69.10 | 18.30 | 25.40 |
| 006 | 70.30 | 39.50 | 25.00 |
| 007 | 64.00 | 34.80 | 25.20 |
| 008 | 62.40 | 32.60 | 21.40 |
| 009 | 69.70 | 38.90 | 29.30 |
| 010 | 47.30 | 23.60 | 13.50 |
| 011 | 63.30 | 34.30 | 22.10 |
| 012 | 69.90 | 38.20 | 26.90 |
| 013 | 78.30 | 45.80 | 25.10 |
| 014 | 58.60 | 30.10 | 22.50 |
| 015 | 68.20 | 36.80 | 23.60 |
| 016 | 55.40 | 28.10 | 23.00 |
| 017 | 68.50 | 38.10 | 22.30 |
| 018 | 52.20 | 25.90 | 18.90 |
| 019 | 65.30 | 35.40 | 19.20 |
| 020 | 57.90 | 30.40 | 22.30 |
| 021 | 62.90 | 33.80 | 22.70 |
| 022 | 76.80 | 45.40 | 27.90 |
| 023 | 63.10 | 33.80 | 20.50 |
| 024 | 63.70 | 33.70 | 20.70 |
| 025 | 43.40 | 21.20 | 16.60 |
| 026 | 72.80 | 12.20 | 24.70 |
| 027 | 42.20 | 41.90 | 25.30 |
| 028 | 61.20 | 31.30 | 14.60 |
| 029 | 61.70 | 32.60 | 21.10 |
| 030 | 44.20 | 21.50 | 15.50 |
| 031 | 66.30 | 36.00 | 27.10 |
| 032 | 73.90 | 40.90 | 20.90 |
| 033 | 70.90 | 40.10 | 24.70 |
| 034 | 64.40 | 34.40 | 29.50 |
| 035 | 69.30 | 38.60 | 27.70 |
| 036 | 77.30 | 45.00 | 28.00 |
| 037 | 75.40 | 44.20 | 26.80 |
| 038 | 69.00 | 38.00 | 22.50 |
| 039 | 69.00 | 38.20 | 14.50 |
| 040 | 79.60 | 47.10 | 23.30 |
| 041 | 65.50 | 35.70 | 20.40 |
| 042 | 70.90 | 39.90 | 28.50 |
| 043 | 54.90 | 27.20 | 13.90 |
| 044 | 68.10 | 37.60 | 29.20 |
| 045 | 47.10 | 23.20 | 9.60 |
| 046 | 64.40 | 34.10 | 23.40 |
| 047 | 81.70 | 50.20 | 22.70 |
| 048 | 72.30 | 40.40 | 25.80 |
| 049 | 55.90 | 28.20 | 18.60 |
| 050 | 69.30 | 37.60 | 23.00 |
| 051 | 49.50 | 24.00 | 19.00 |
| 052 | 73.40 | 40.00 | 20.50 |
